# Synthesis, X-ray Crystal Structure, and Photochromism of a Sandwich-Type Mono-Aluminum Complex Composed of Two Tri-Lacunary α-Dawson-Type Polyoxotungstates

**DOI:** 10.3390/ma12152383

**Published:** 2019-07-26

**Authors:** Chika Nozaki Kato, Daichi Kato, Toshifumi Kashiwagi, Shunpei Nagatani

**Affiliations:** 1 Department of Chemistry, Shizuoka University, 836 Ohya, Suruga-ku, Shizuoka 422-8529, Japan; 2 Research Institute of Green Science and Technology, Shizuoka University, 836 Ohya, Suruga-ku, Shizuoka 422-8529, Japan

**Keywords:** polyoxometalate, aluminum complex, ion-exchange resin, X-ray crystallography, photochromic property

## Abstract

The synthesis and molecular structure of a dimeric, mono-aluminum complex composed of two tri-lacunary α-Dawson polyoxometalates, [H_14_Al(B-α-P_2_W_15_O_56_)_2_]^7−^ (1), is described herein. The tetra-*n*-butylammonium salt of 1, [(*n*-C_4_H_9_)_4_N]_7_[H_14_Al(B-α-P_2_W_15_O_56_)_2_] (TBA-1) was prepared by passing an aqueous solution of K_6_[B-α-H_3_P_2_W_15_O_59_{Al(OH_2_)}_3_]⋅14H_2_O through an ion-exchange resin column (H^+^-form), followed by addition of tetra-*n*-butylammonium bromide. Analytically pure and colorless crystals of TBA-1 were obtained via vapor diffusion from acetonitrile/methanol at ~25 °C. Single-crystal X-ray structure analysis revealed that a six-coordinate aluminum ion was sandwiched between two tri-lacunary α-Dawson-type units, resulting in an overall *C*_2*h*_ symmetry. The characterization of TBA-1 was accomplished by elemental analyses, thermogravimetric/differential thermal analyses, Fourier-transform infrared spectroscopy, and solution ^31^P nuclear magnetic resonance spectroscopy. The photochromic properties of TBA-1 were also characterized in methanol under light irradiation (λ = 365 nm and ≥400 nm).

## 1. Introduction

Aluminum and its derivative alloys, oxides, organometallics, and inorganic compounds have been used in many fields due to their unique properties including high reactivity, acidity, hardness, and electroconductivity [1,2]. Since the properties and activities of aluminum species strongly depend on the aluminum site structures, the synthesis of aluminum compounds with structurally well-defined aluminum sites is important for the development of useful aluminum-based materials. However, even when conditions are carefully controlled during preparation, the construction of well-defined aluminum structure is often difficult [2,3,4].

Polyoxometalates have attracted much attention in the fields of catalytic chemistry, surface science, and materials science because of their controllable shape, size, composition, and structural diversity [5,6,7,8,9]. Although various techniques for functionalization of polyoxometalates have been reported, the synthetic technology of introducing metal ions into vacant sites using lacunary polyoxometalates as inorganic ligands is an effective technique for constructing stable and well-defined active metal centers. In terms of polyoxometalates containing aluminum ions as heteroatoms, various aluminum compounds have been synthesized and structurally characterized [10,11,12,13,14,15,16,17]. Although some aluminum-containing polyoxometalates, e.g., [γ-SiW_10_O_36_{Al(OH_2_)}_2_(μ-OH)_2_]^4−^ [10], [(A-PW_9_O_34_)_2_{W(OH)(OH_2_)}{Al(OH)(OH_2_)}{Al(μ-OH)(OH_2_)_2_}_2_]^7−^ [11], [α-PW_11_{Al(OH_2_)}O_39_]^4−^ [11], and [α_2_-P_2_W_17_{Al(OH_2_)}O_61_]^7−^ [11], and [Al_4_(H_2_O)_10_(β-AsW_9_O_33_H)_2_]^4−^ [12], have been used as Lewis acids and oxidation catalysts, examples of structurally characterized aluminum complexes with polyoxometalate ligands remain rare, and their properties and activities are unknown.

Herein, we successfully obtained a dimeric, mono-aluminum complex composed of tri-lacunary α-Dawson polyoxometalate units in the form of crystals suitable for the X-ray structure analysis of [(*n*-C_4_H_9_)_4_N]_7_[H_14_Al(B-α-P_2_W_15_O_56_)_2_] (TBA-1). Polyoxoanion 1 exhibited excellent photochromic properties both in the presence of methanol or ethanol in solutions of acetonitrile and DMSO and in suspension under light irradiation (λ = 365 nm and ≥400 nm). Although the photochromic behavior of polyoxometalate is routinely observed, there are few examples of photoresponsive materials obtained by transformation of polyoxometalate structures without including photoresponsive organic molecules [18,19]. We report the synthesis, X-ray crystal structure, and photochromic properties of TBA-1.

## 2. Experimental

### 2.1. Materials and Methods

K_6_[B-α-H_3_P_2_W_15_O_59_{Al(OH_2_)}_3_]⋅14H_2_O was prepared as previously described [13]. The number of solvated water molecules was determined by thermogravimetric/differential thermal analyses (TG/DTA). All reagents and solvents were obtained and used as-received from commercial sources. The elemental analyses results of C, H, and N were obtained using Flash EA (Thermo Electron Corporation, Waltham, MA, USA) at Shizuoka University (Shizuoka, Japan). The elemental analyses of P, Al, and K were performed by Mikroanalytisches Labor Pascher (Remagen, Germany). Fourier-transform infrared (FT-IR) spectra were recorded using a Perkin Elmer Spectrum 100 FT-IR spectrometer (Waltham, MA, USA) on KBr disks at ~25 °C. The TG/DTA data were obtained using Rigaku Thermo Plus EVO2 TG/DTA 81205Z instrument (Tokyo, Japan) in air while increasing the temperature from 20 to 500 °C at 4 °C/min. The ^31^P (242.95 MHz) NMR spectrum in solution was recorded in 5 mm outer diameter tubes using a JEOL ECA-600 NMR spectrometer (Akishima, Tokyo) at Shizuoka University. The ^31^P NMR spectra were measured in DMSO-*d*_6_ with reference to an external standard of 85% H_3_PO_4_ in a sealed capillary. The chemical shifts were reported as negative on the δ scale for resonances upfield of H_3_PO_4_ (δ 0). For the photochromism experiments, the crystals of TBA-1 were dissolved in a DMSO/methanol (83:17 vol%) solution, and the solution was irradiated in a quartz cell with a 300 W Xe lamp (λ = ≥400 and ≥440 nm) or 6W Hg lamp (λ = 254 and 365 nm). All measurements were performed under ambient conditions. The UV–Vis spectra were recorded using a Perkin–Elmer Spectrum Lambda 650 spectrophotometer. The experiment of coloration–decoloration cycles was carried out as follows: the DMSO/methanol (83:17 vol%) solution of TBA-1 (6.6 × 10^−4^ M) was irradiated by light (λ = ≥400 nm) for 30 min in air, and UV–Vis spectrum was immediately observed. After stand for several hours in the dark, the UV–Vis spectrum was observed again. This cycle was repeated several times, and the absorbance at 655 nm was plotted.

### 2.2. Synthesis of [(n-C_4_H_9_)_4_N]_7_[H_14_Al(B-α-P_2_W_15_O_56_)_2_] (TBA-1)

K_6_[B-α-H_3_P_2_W_15_O_59_{Al(OH_2_)}_3_]⋅14H_2_O (5.047 g, 1.15 mmol) was dissolved in 130 mL of water in a water bath at approximately 90 °C. After being cooled to approximately 25 °C, the colorless clear solution was passed through a cation exchange resin column (Amberlite IR120B NA, 163 mL) at a rate of 1 drop / s. Subsequently, 250 mL of water was passed through the column. It was confirmed that the pH of the eluent was the same as that of the water. Solid [(*n*-C_4_H_9_)_4_N]Br (33.376 g, 0.104 mol) was then added to the aqueous solution. After stirring overnight, white precipitate was collected using a glass flit (17G4) then washed with water (50 mL × 3) and ethanol (50 mL × 3). The crude product was obtained in a 5.330 g yield. For purification, the crude product (2.00 g) was dissolved in 20 mL of acetonitrile, and the insoluble white precipitate was removed using a folded filter paper (Whatman #5). Crystallization was performed by vapor diffusion from methanol at approximately 25 °C. After standing for a few days, colorless crystals were collected by a membrane filter (JG 0.2 µm; note: the crystals of TBA-1 changed from white to blue during crystallization under fluorescent light, but returned to white when contact with methanol vapor was stopped).

The product was obtained in a yield of 0.739 g (the yield calculated based on [mol of TBA-1]/[mol of K_6_[B-α-H_3_P_2_W_15_O_59_{Al(OH_2_)}_3_]⋅14H_2_O] × 100 was 18.7%). A single crystal for X-ray structural analysis was prepared by vapor diffusion from methanol using 0.500 g of the obtained product. The elemental analysis results showed C, 14.97; H, 2.84; Al, 0.31; N, 1.25; P, 1.35; K, <0.01%, and calculations for [(*n*-C_4_H_9_)_4_N]_7_[H_14_Al(B-α-P_2_W_15_O_56_)_2_] (TBA-1) = C_112_H_266_Al_1_N_7_O_112_P_4_W_30_ (MW 9169.4) showed C, 14.67; H, 2.92; Al, 0.29; N, 1.07; P, 1.35; K, 0%. The TG/DTA data under atmospheric conditions showed a weight loss of 17.5% with exothermic peaks at 320.0 and 465.8 °C from 25.4 to 465.8 °C, whereas calculations showed a 18.5% weight loss for seven tetra-*n*-butylammonium ions. The IR (KBr disk) results in the 1300 to 400 cm^−1^ region (polyoxometalate region) showed bands at 1091, 1052, 998, 966, 952, 917, 790, 599, and 532 cm^−1^. ^31^P NMR: (DMSO-*d*_6_ with a drop of water, 23.8 °C): δ −6.36, −11.80.

### 2.3. X-ray Crystallography

A colorless block crystal of TBA-1 (0.080 mm × 0.080 mm × 0.050 mm) was mounted on a MicroMount. The measurements were performed using a Rigaku VariMax with an XtaLAB P200 diffractometer (Rigaku Inc., Tokyo, Japan) using multilayer mirror-monochromated Mo Kα radiation (λ = 0.71075 Å) at 153 ± 1 K. The data were collected and processed using CrystalClear (Rigaku Inc., 2008), CrystalClear-SM Expert for Windows (Rigaku Inc., 2008), and structural analysis was performed using CrystalStructure for Windows (Version 4.2.5). The structure was solved using SHELXS-2013 and refined by SHELXL-2016 [20]. For polyoxoanion 1, 30 tungsten atoms, an aluminum atom, four phosphorus atoms, and 112 oxygen atoms were identified. However, the resolution obtained for the structure was limited by the poor quality of the available crystals and considerable disorder of the counter cations, which are common in polyoxometalate crystallography [21,22,23,24,25]. Accordingly, the residual electron density was removed using the SQUEEZE [26] routine in PLATON (Version 1.17, University of Glasgow, Scotland, UK).

### 2.4. Crystal Data of TBA-1

C_112_H_266_Al_1_N_7_O_112_P_4_W_30_; MW = 9169.4, orthorhombic, space group: *Cmce* (#64), *a* = 25.861(3) Å, *b* = 17.413(2) Å, *c* = 52.237(7) Å, *V* = 23,524(5) Å^3^, *Z* = 4, *D*_c_ = 2.589 g/cm^3^, μ(Mo Kα) = 147.274 cm^−1^, *R*_1_ = 0.0816 [*I* > 2σ(*I*)], *wR*_2_ = 0.2407 (for all data). GOF = 0.984 (66,740 total reflections and 10,464 unique reflections where *I* > 2σ(*I*)). CSD No. 1920906.

## 3. Results and Discussion

### 3.1. Synthesis and Characterization of [(n-C_4_H_9_)_4_N]_7_[H_14_Al(B-α-P_2_W_15_O_56_)_2_] (TBA-1)

The tetra-*n*-butylammonium salt of a mono-aluminum complex with two tri-lacunary α-Dawson-type polyoxotungstes, [(*n*-C_4_H_9_)_4_N]_7_[H_14_Al(B-α-P_2_W_15_O_56_)_2_] (TBA-1), was prepared by passing the aqueous solution of the monomeric, α-Dawson-type tri-aluminum-substituted polyoxotungstate, K_6_[B-α-H_3_P_2_W_15_O_59_{Al(OH_2_)}_3_]⋅14H_2_O, through an ion exchange resin column (H^+^-form), followed by addition of tetra-*n*-butylammonium bromide. The purification was performed by crystallization via vapor diffusion from acetonitrile/methanol at 25 °C in air. TBA-1 was finally isolated as an analytically pure, colorless crystals in a 18.7% yield. 

Here, the molecular structure of α-Dawson-type tri-aluminum-substituted polyoxotungstate used as the starting complex changed as it passed through the ion exchange resin (H^+^-form). In general, ion exchange resins are used to obtain the free-acid type of polyoxometalates by the exchange of counter cations (including alkali metal ions and organic ammonium ions) with protons [27,28]. However, this treatment of α-Dawson-type tri-aluminum-substituted polyoxotungstate with ion exchange resin caused the cation exchange reaction and the molecular structure transformation of a monomeric, α-Dawson-type tri-aluminum-substituted polyoxotungstate to a dimeric, α-Dawson-type mono-aluminum-substituted polyoxotungstate. Although the formation of polyoxoanion 1 was observed by adjusting the pH of K_6_[B-α-H_3_P_2_W_15_O_59_{Al(OH_2_)}_3_]⋅14H_2_O aqueous solution to approximately 0.05; TBA-1 with high purity was obtained by passing through the ion-exchange resin column (H^+^-form).

The elemental analyses of C, H, N, Al, and P were in good agreement with the calculated values for the formula without hydrated water molecules for TBA-1 (see the Experimental Section). It should be noted that the K analysis revealed no potassium ion contamination in the precursor. For the TG/DTA measurement performed under atmospheric conditions, a weight loss of 17.5% was observed at 25.4–465.8 °C corresponding to seven tetra-n-butylammonium ions (calcd. 18.5%), as shown in Figure S1.

**Figure 1:**
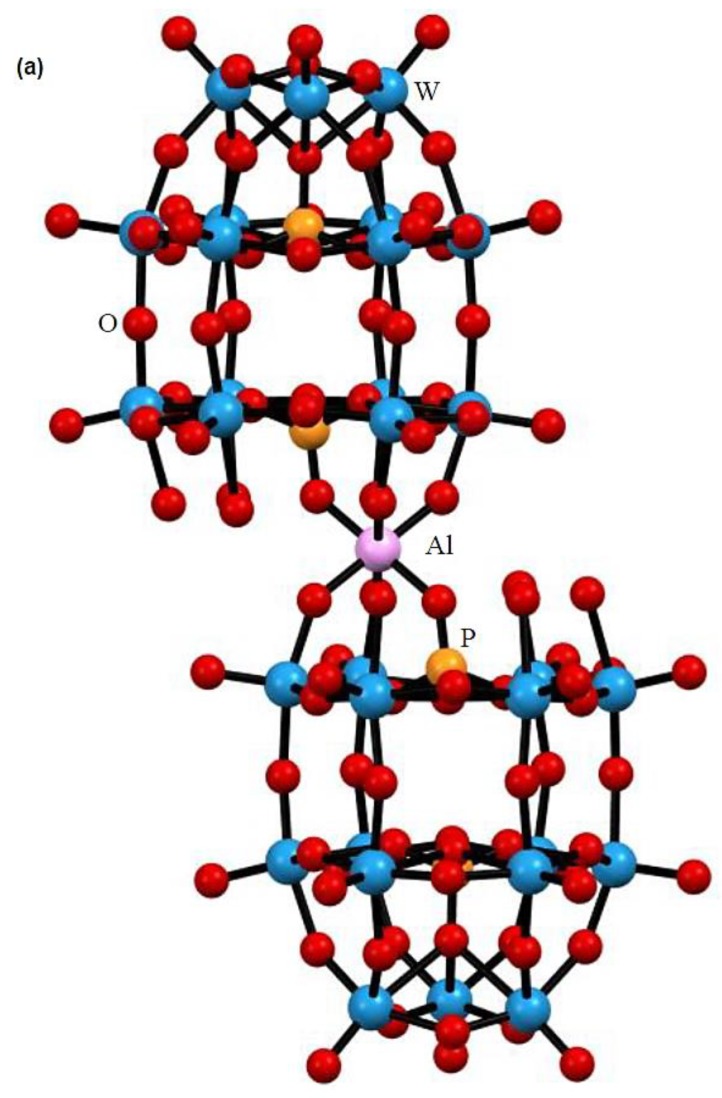

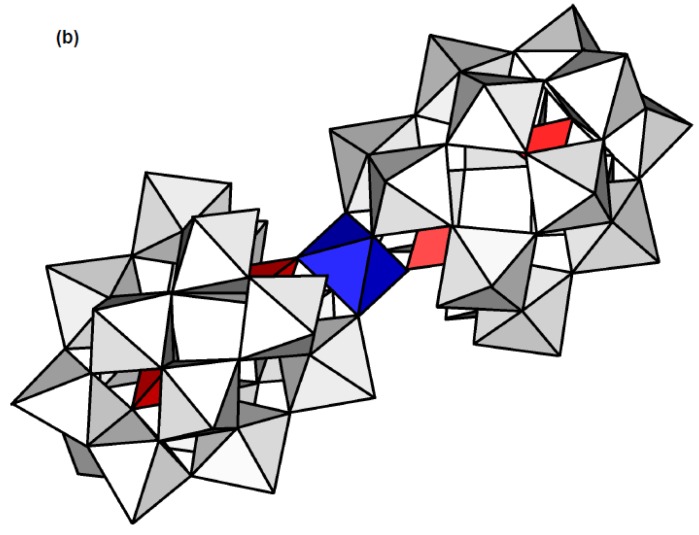
(**a**) Molecular structure (ball and stick representation) and (**b**) polyhedral representation of the polyoxoanion [H_14_Al(B-α-P_2_W_15_O_56_)_2_]^7^^−^ (1). In the polyhedral representation, the AlO_6_, WO_6_, and internal PO_4_ groups are represented blue and white octahedra, and red tetrahedra, respectively.

Single crystals of TBA-1 suitable for X-ray crystallography were obtained by crystallization via vapor diffusion from acetonitrile/methanol. The molecular structure of [H_14_Al(B-α-P_2_W_15_O_56_)_2_]^7^^−^ (1) in TBA-1 and a Dawson unit with atom numbering are shown in Figure 1 and Figure S2, respectively. The bond lengths and angles are listed in Tables S1 and S2. X-ray crystallography of 1 revealed that a 6-coordinate mono-aluminum site was sandwiched by two tri-lacunary α-Dawson polyoxotungstate units, [α-P_2_W_15_O_56_]^12−^, resulting in an overall *C*_2*h*_ symmetry. Similar dimeric structure coexisting with substitution sites and noncoordinating terminal oxo groups between the two {P_2_W_15_} units was previously reported for [(TiO_2_W_15_O_55_H)_2_]^14−^ [29], [Ti_2_{P_2_W_15_O_54_(OH_2_)_2_}_2_]^8−^ [27], and [{P_2_W_15_O_54_(H_2_O)}_2_Zr]^12−^ [30], but TBA-1 is the first example of a compound containing an aluminum site.

The bond valence sums (BVSs) [31,32,33,34], calculated based on the observed bond lengths for TBA-1, ranged from 5.65 to 6.45 (average of 6.06) for the eight W atoms, 5.2–5.49 (average of 5.36) for the two P atoms, 1.52–2.80 (average of 1.95) for the 31 oxygen atoms (excluding O(9) and O(10)), and 2.88 for the Al atom (Table S3). These values were consistent with the formal valences of W^6+^, P^5+^, O^2−^, and Al^3+^. In contrast, the calculated BVS values of the terminal oxygen atoms at the vacant sites were 0.54 for O(9) and 0.63 for O(10). These BVS values of the oxygen atoms suggested that one or two protons were bound to each terminal oxygen atoms, as reported for K_6_Na[(A-PW_9_O_34_)_2_{W(OH)(OH_2_)}{Al(OH)(OH_2_)}{Al(µ-OH)(OH_2_)_2_}_2_]⋅19H_2_O [15] and [(CH_3_)_4_N]_14_Na_2_[B-α-H_3_P_2_W_15_O_59_{Al(OH)}_2_{Al(OH_2_)}]_2_⋅39H_2_O [13]. Thus, the elemental analyses and BVS calculations suggested that 14 protons were present as hydroxyl groups and/or water molecules at the vacant sites.

**Figure 2:**
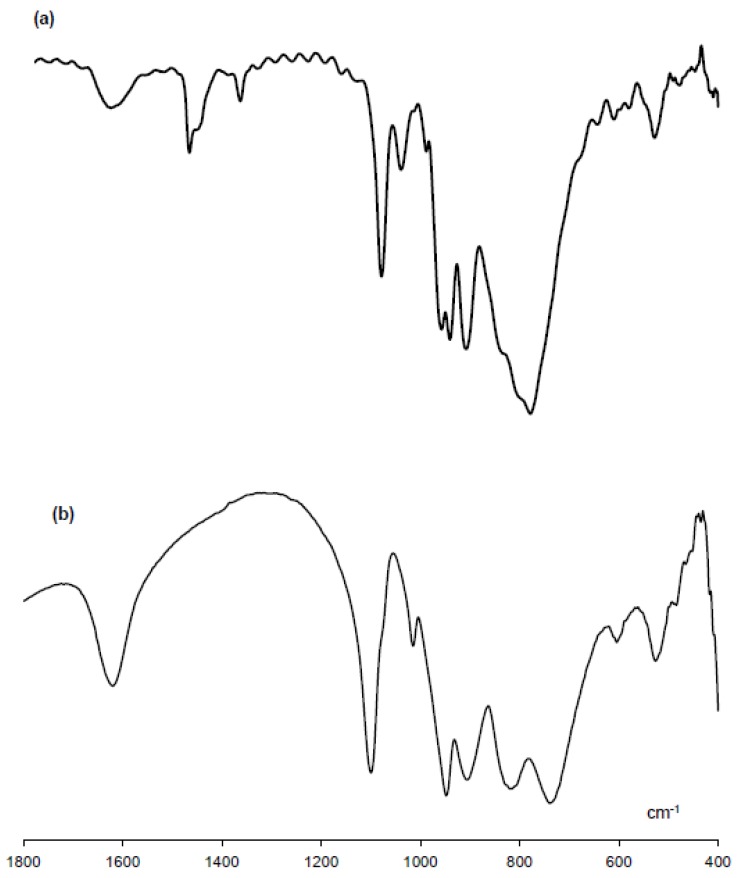
FT-IR spectra in the polyoxoanion region (1800–400 cm^−1^), measured as KBr disks, of (**a**) TBA-1 and (**b**) K_6_[B-α-H_3_P_2_W_15_O_59_{Al(OH_2_)}_3_]⋅14H_2_O.

The FT-IR spectrum measured as a KBr disk for TBA-1 is shown in Figure 2. The spectral pattern of TBA-1 (bands at 1091, 1052, 998, 966, 952, 917, 790, 599, and 532 cm^−1^; Figure 2a) was different from that of the starting material K_6_[B-α-H_3_P_2_W_15_O_59_{Al(OH_2_)}_3_]⋅14H_2_O (bands at 1100, 1015, 948, 906, 820, 739, 605, and 526 cm^−1^; Figure 2b), the α-Dawson-type tri-lacunary polyoxometalate Na_12_[B-α-P_2_W_15_O_56_]⋅33H_2_O (bands at 1132, 1087, 1009, 978, 937, 915, 876, 826, 744, and 526 cm^−^^1^) [13,35], α_2_-Dawson-type mono-lacunary polyoxometalate K_10_[α_2_-P_2_W_17_O_61_]·23H_2_O (bands at 1631, 1082, 1050, 1017, 940, 922, 889, 817, 748, and 528 cm^−^^1^) [36], and α_2_-Dawson-type mono-aluminum-substituted polyoxometalate K_7_[α_2_-P_2_W_17_{Al(OH_2_)}O_61_]⋅14H_2_O (bands at 1090, 1018, 952, 916, 796, and 526 cm^−^^1^) [11]. This suggested that the molecular structure of [B-α-H_3_P_2_W_15_O_59_{Al(OH_2_)}_3_]^6^^−^ changed to a new species as it was passed through the ion exchange resin. The band at 1484 cm^−1^ arose from the tetra-*n*-butylammonium ions.

**Figure 3:**
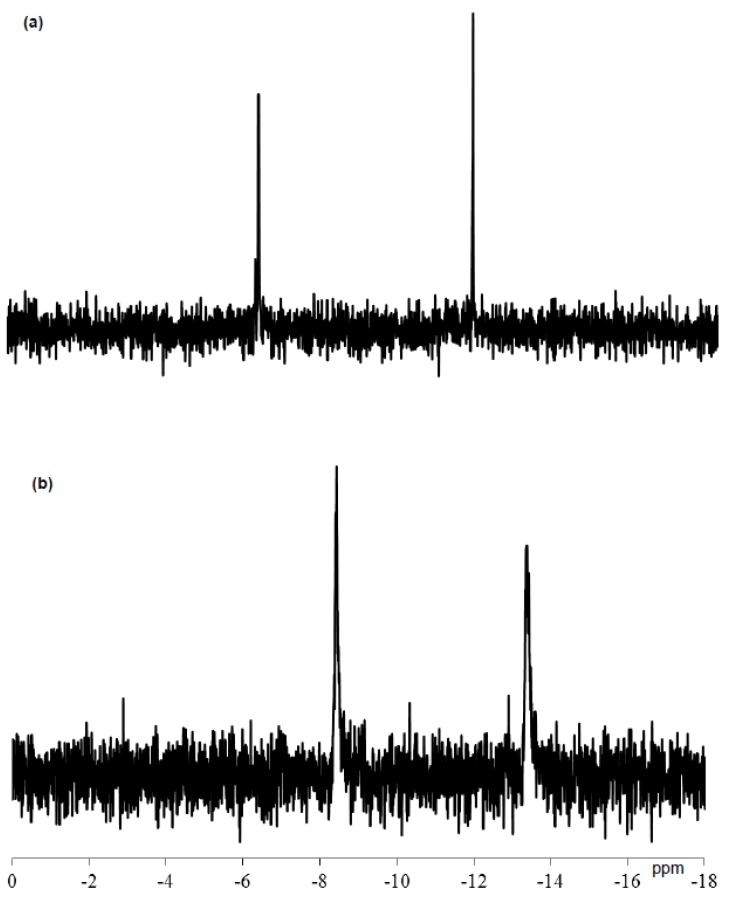
^31^P NMR spectra in DMSO-d_6_ of (**a**) as-prepared TBA-1 in the presence of a drop of water and (**b**) K_6_[B-α-H_3_P_2_W_15_O_59_{Al(OH_2_)}_3_]⋅14H_2_O (δ −8.43, −13.39) in an 11:2 (v/v%) DMSO-d_6_/water solution. The spectrum was referenced to an external standard of 85% H_3_PO_4_ in a sealed capillary.

The ^31^P NMR spectrum of TBA-1 in DMSO-d_6_ containing a drop of water at 23.8 °C showed two main signals at −6.36 and −11.80 ppm with approximately 1:1 integrated intensities arising from the two internal phosphorus atoms, as shown in Figure 3a. The signals differed from those of K_6_[B-α-H_3_P_2_W_15_O_59_{Al(OH_2_)}_3_]⋅14H_2_O (δ −8.43, −13.39) observed in 11:2 (v/v%) DMSO-d_6_/water (Figure 3b), suggesting the formation of a novel polyoxometalate-based species.

### 3.2. Photochromism of TBA-1

During crystallization of TBA-1 from acetonitrile/methanol, we coincidentally observed the photochromic behavior of TBA-1 under light (λ = 365 nm and ≥400 nm) irradiation in the presence of alcohol (methanol or ethanol) in suspension and solution. When solid TBA-1 was suspended in methanol and irradiated by the light for a few minutes, the solid TBA-1 turned from white to blue. When the compound was left for several hours in the dark, it returned to white under an air. In DMSO/methanol solution, TBA-1 also showed similar photochromic properties under light irradiation (λ = 365 nm and ≥400 nm) within a few minutes. Under UV (λ = 254 nm) and visible light (λ = ≥440 nm) irradiation, the coloration was not observed within at least a few hours. 

**Figure 4:**
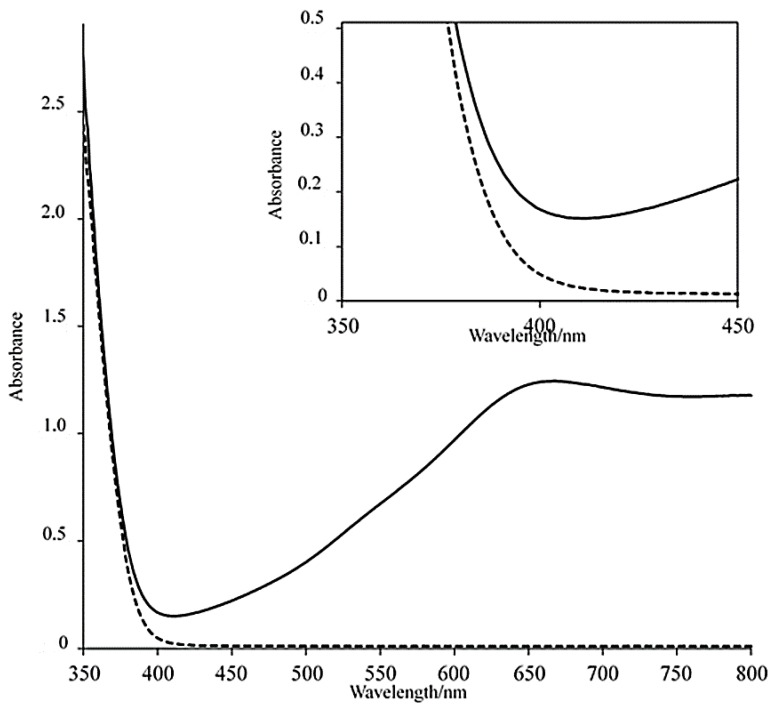
UV–Vis spectra from 350 to 800 nm of TBA-1 (6.6 × 10^−4^ M) in DMSO/methanol (83:17 vol%) solution before (dashed line) and after 30 min of light irradiation (solid line). In set: the absorption at 350–450 nm.

Figure 4 shows the UV–Vis spectra from 350 to 800 nm of TBA-1 in the DMSO/methanol (83:17 vol%) solution before and after 30 min of photoirradiation at ≥400 nm. In the spectrum before light irradiation, an absorption tail was red-shifted to approximately 400 nm as compared to the absorption in the absence of methanol (Figure S3). The red-shift of absorption tail due to the presence of organic molecules was also observed in H_3_PMo_12_O_40_⋅6DMA⋅CH_3_CN⋅0.5H_2_O (DMA = *N*,*N*-dimethylacetamide), which is highly photosensitive in the near-UV and visible region [37]. Similar behavior was observed for H_3_PW_12_O_40_ [38] and H_4_SiW_12_O_40_ [39]. The electronic interactions between TBA-1 and methanol (or ethanol) are essential for the red-shifting of the O→M LMCT band, allowing photochromism to be initiated by visible light (λ = ≥400 nm) irradiation [18]. After irradiation for 30 min, a large absorption with a maximum at approximately 655 nm was observed, suggesting that the tungsten sites in the {P_2_W_15_} units were reduced to heteropolyblue species [5,40]. The adsorption completely disappeared when the solution was shielded from light under an air atmosphere for several hours. With a few minutes of argon purge in an airtight vial, the color did not return from blue to white for at least several days. These results suggested that the heteropolyblue species was reoxidized by oxygen in air.

Despite the presence of 365 nm absorption, TBA-1 did not show any distinct coloration under UV light irradiation (λ = 365 nm) in the absence of methanol (or ethanol). These results suggested that the presence of methanol (or ethanol) was essential for the photoreduction of TBA-1 under UV light (λ = 365 nm) as well as visible light (λ = ≥400 nm) irradiation. When a small portion of water was added to the DMSO/methanol solution of TBA-1, the photochromic properties were no longer expressed, likely because of the inhibition of methanol contact by water. This was also supported the result that the absorption at around 400 nm was blue-shifted by the addition of water to the DMSO/methanol solution, as shown in Figure S4.

**Figure 5:**
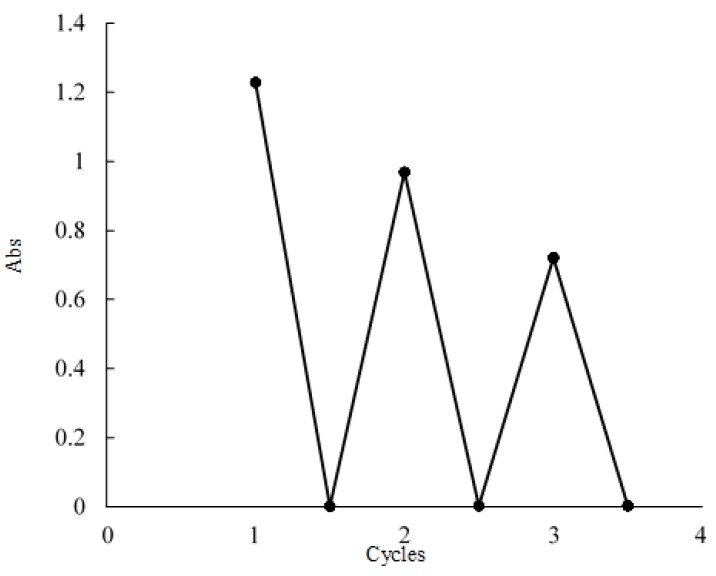
Coloration–decoloration cycles of TBA-1 in DMSO/methanol (83:17 vol%) solution, monitored by absorbance at 655 nm.

**Figure 6:**
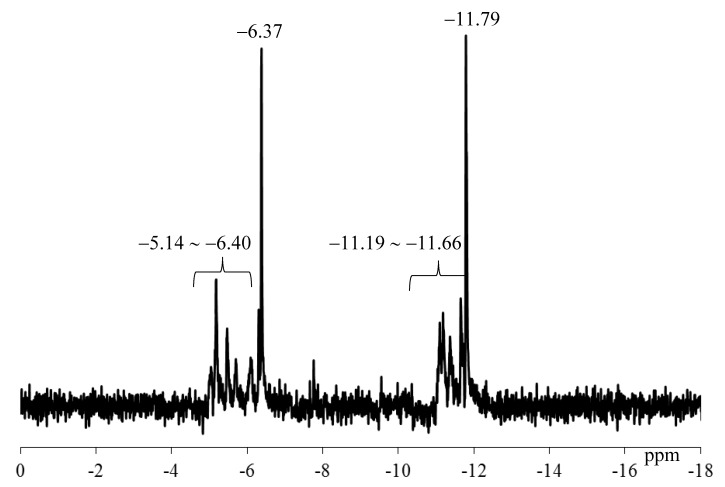
^31^P NMR spectrum in DMSO-d_6_ of TBA-1 in the presence of a drop of water after standing for six days in DMSO-d_6_ at approximately 25 °C. The spectrum was referenced to an external standard of 85% H_3_PO_4_ in a sealed capillary.

With successive coloration–decoloration cycles, the position of absorption band was largely unchanged and the photochromic reversibility of TBA-1 was confirmed in DMSO/methanol (83:17 vol%). However, the absorbance of the band at 655 nm arising from the reduced species gradually decreased with the number of cycles, as shown in Figure 5. The ^31^P NMR spectrum of TBA-1 after standing for 6 days in DMSO-d_6_ at approximately 25 °C is shown in Figure 6. The signals of the as-prepared TBA-1 were retained at −6.37 and −11.79 ppm, but many unassigned signals were also observed. These results suggest that the decreased absorption was due to the decomposition of TBA-1 in the DMSO solution.

Although the stability of TBA-1 in DMSO solution was only moderate, the polyoxometalate compounds exhibiting visible-light coloration are useful materials [18,19]. To the best of our knowledge, TBA-1 is the first example of a photoresponsive aluminum-containing polyoxometalate.

## 4. Conclusions

Herein, a dimeric aluminum complex composed of tri-lacunary α-Dawson polyoxometalate was prepared. Single crystals of a tetra-*n*-butylammonium salt [(*n*-C_4_H_9_)_4_N]_7_[H_14_Al(B-α-P_2_W_15_O_56_)_2_] (TBA-1) were obtained by passing the aqueous solution of monomeric, α-Dawson tri-aluminum-substituted polyoxometalate K_6_[B-α-H_3_P_2_W_15_O_59_{Al(OH_2_)}_3_]⋅14H_2_O through an ion exchange resin column (H^+^-form), followed by addition of tetra-*n*-butylammonium bromide. The characterization of TBA-1 was accomplished by X-ray structure analysis, elemental analyses, TG/DTA, FT-IR, and solution ^31^P NMR spectroscopy. The single-crystal X-ray structure revealed that a six-coordinate aluminum ion was sandwiched between two tri-lacunary α-Dawson-type units, resulting in an overall *C*_2*h*_ symmetry. Surprisingly, TBA-1 exhibited photochromic properties in the presence of alcohol (methanol or ethanol) in solutions of acetonitrile and DMSO and in suspension under UV (λ = 365 nm) and visible light irradiation (λ = ≥400 nm). Although the photochromic behavior of polyoxometalate is routinely observed, TBA-1 is a rare example of photoresponsive materials obtained by transformation of polyoxometalate structures. In future studies, we will investigate the effect of polyoxometalate constituents (e.g., aluminum sites, polyoxometalate structures, and counter cations) on these photochromic properties.

## Supplementary Materials

The following are available online at https://www.mdpi.com/1996-1944/12/15/2383/s1. Figure S1: TG/DTA data of TBA-1; Figure S2: A Dawson unit of [H_14_Al(B-α-P_2_W_15_O_56_)_2_]^7−^ (1) with atom numbering; Figure S3: UV–Vis spectrum at 350–800 nm of TBA-1 in DMSO with a small portion of water; Figure S4: UV–Vis spectrum at 300–460 nm of TBA-1 in DMSO/methanol (83:17 vol%) solution and in DMSO/methanol/water (75:10:15 vol%) solution; Table S1: Bond length (Å) of TBA-1; Table S2: Bond angles (°) of TBA-1; Table S3: Bond valence sums (BVSs) of polyoxoanion 1.
