# Supplementary material for: Synthesis, X-ray Crystal Structure, and Photochromism of a Sandwich-Type Mono-Aluminum Complex Composed of Two Tri-Lacunary α-Dawson-Type Polyoxotungstates
Source: Materials (Basel). 2019 Jul 26;12(15):2383. doi: 10.3390/ma12152383 (PMC6696191; doi:10.3390/ma12152383)
Supplement: Supplementary file 1 [file materials-12-02383-s001.pdf]

materials

Supplementary Materials: Synthesis, X-ray Crystal Structure, and Photochromism of a Sandwich-Type Mono-Aluminum Complex Composed of Two Tri-lacunary α-Dawson-Type Polyoxotungstates

Chika Nozaki Kato, Daichi Kato, Toshifumi Kashiwagi and Shunpei Nagatani

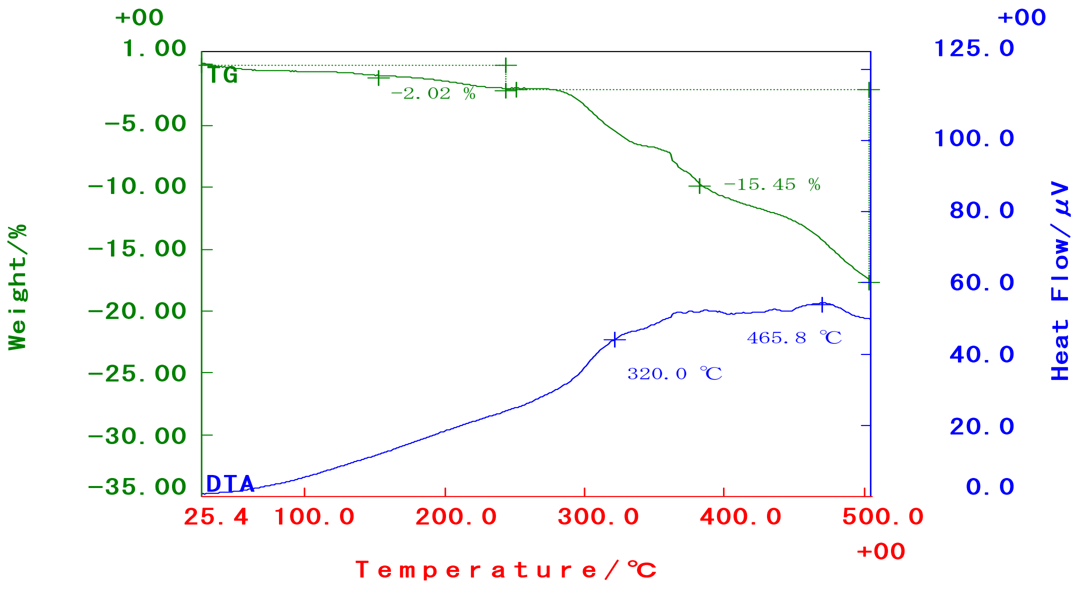

Figure S1. TG/DTA data of TBA-1.

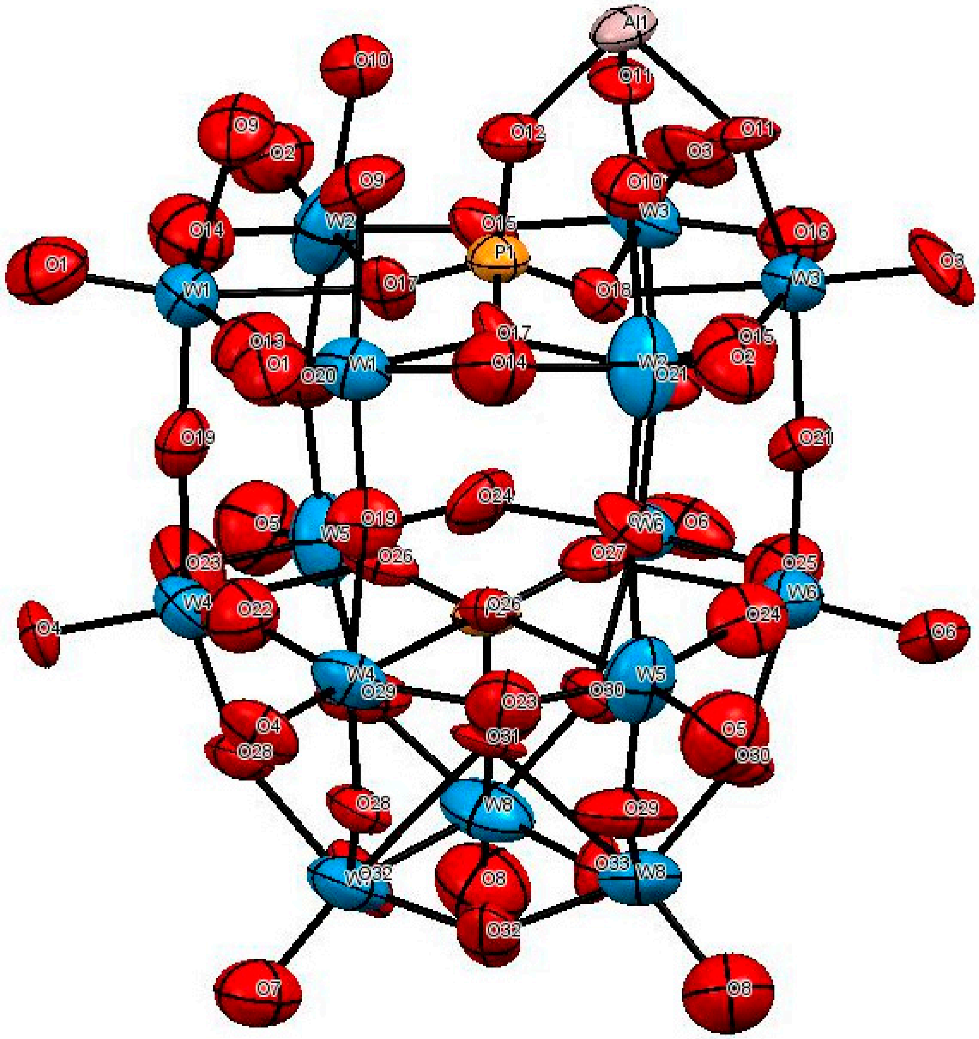

Figure S2. A Dawson unit of $[H_{14}Al(B\text{-}\alpha\text{-}P_2W_{15}O_{56})_2]^{7-}$ (**1**) with atom numbering.

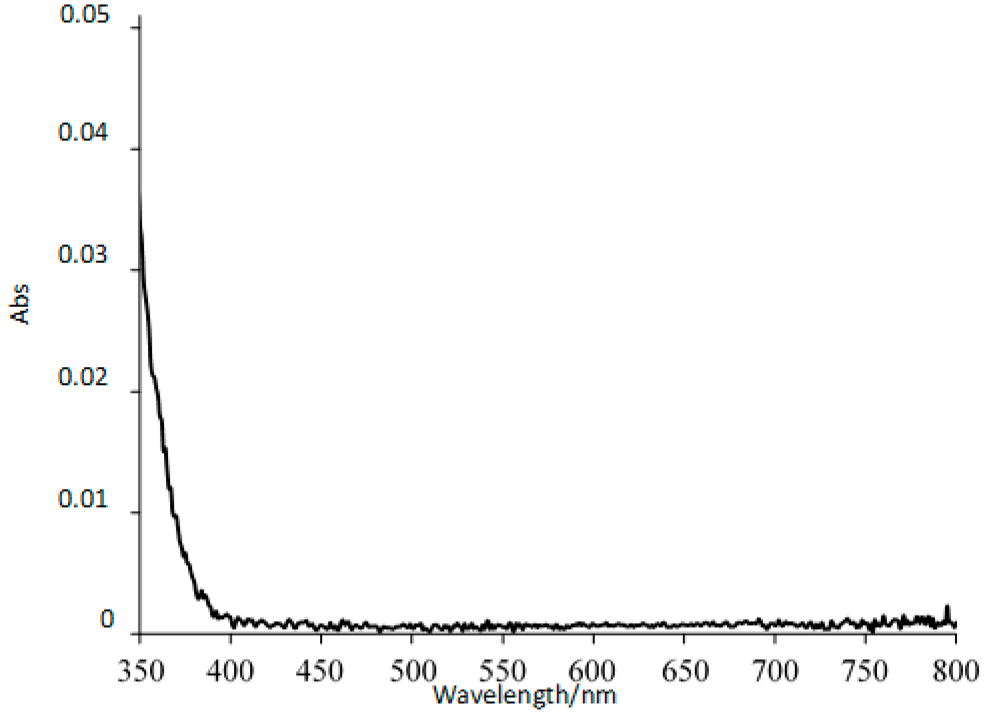

Figure S3. UV-vis spectrum at 350 – 800 nm of **TBA-1** in DMSO with a small portion of water.

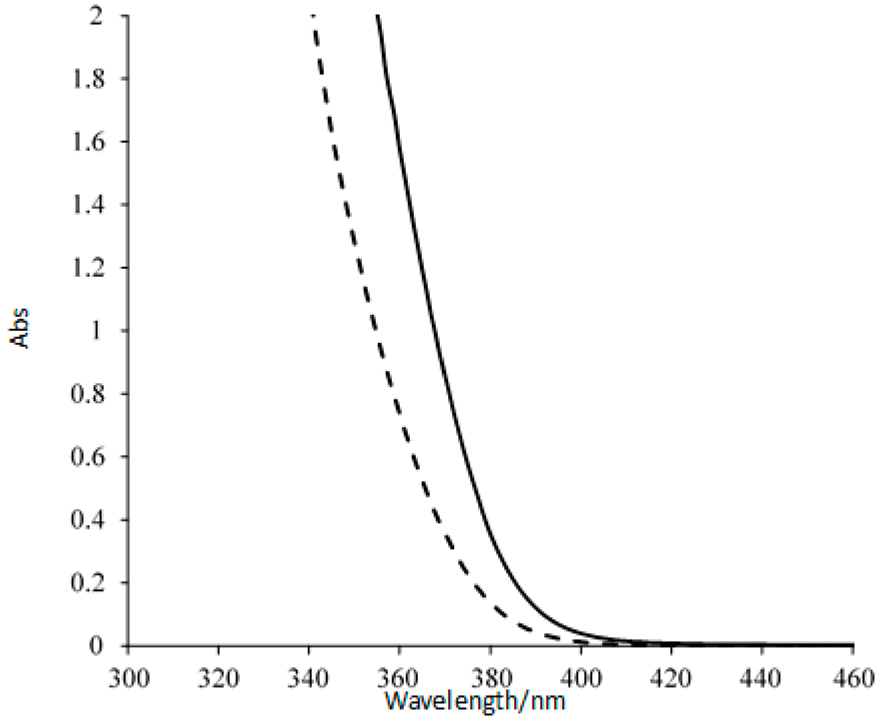

Figure S4. UV-vis spectrum at 300 – 460 nm of **TBA-1** in DMSO/methanol (83:17 vol%) solution (solid line) and in DMSO/methanol/water (75:10:15 vol%) solution (dashed line).

Table S1. Bond length (Å) of **TBA-1.**

**Bond length (Å)**			
W(1)-O(1)	1.690(14)	W(1)-O(9)	2.143(15)
W(1)-O(13)	1.874(3)	W(1)-O(14)	1.818(19)
W(1)-O(17)	2.431(11)	W(1)-O(19)	1.838(13)
W(2)-O(2)	1.708(19)	W(2)-O(10)	2.104(12)
W(2)-O(14)	1.970(17)	W(2)-O(15)	1.854(13)
W(2)-O(17)	2.330(11)	W(2)-O(20)	1.866(10)
W(3)-O(3)	1.762(16)	W(3)-O(11)	1.895(11)
W(3)-O(15)	1.896(12)	W(3)-O(16)	1.972(10)
W(3)-O(18)	2.357(10)	W(3)-O(21)	1.982(13)
W(4)-O(4)	1.758(12)	W(4)-O(19)	1.948(13)
W(4)-O(22)	1.905(6)	W(4)-O(23)	1.991(15)
W(4)-O(26)	2.385(11)	W(4)-O(28)	1.871(11)
W(5)-O(5)	1.71(2)	W(5)-O(20)	1.944(11)
W(5)-O(23)	1.857(15)	W(5)-O(24)	1.903(13)
W(5)-O(26)	2.436(12)	W(5)-O(29)	1.891(15)
W(6)-O(6)	1.727(16)	W(6)-O(21)	1.840(12)
W(6)-O(24)	1.909(13)	W(6)-O(25)	1.943(9)
W(6)-O(27)	2.387(11)	W(6)-O(30)	1.993(11)
W(7)-O(7)	1.68(2)	W(7)-O(28)	1.980(12)
W(7)-O(28)^{1}	1.980(12)	W(7)-O(31)	2.394(12)
W(7)-O(32)	1.821(11)	W(7)-O(32)^{1}	1.821(11)
W(8)-O(8)	1.72(2)	W(8)-O(29)	1.967(17)
W(8)-O(30)	1.839(11)	W(8)-O(31)	2.418(12)
W(8)-O(32)	1.972(11)	W(8)-O(33)	1.793(4)
P(1)-O(12)	1.443(17)	P(1)-O(17)	1.559(11)
P(1)-O(17)^{1}	1.559(11)	P(1)-O(18)	1.526(16)
P(2)-O(26)	1.465(13)	P(2)-O(26)^{1}	1.465(13)
P(2)-O(27)	1.542(17)	P(2)-O(31)	1.536(14)
Al(1)-O(11)	1.900(12)	Al(1)-O(11)^{2}	1.900(12)
Al(1)-O(11)^{3}	1.900(12)	Al(1)-O(11)^{1}	1.900(12)
Al(1)-O(12)	1.876(16)	Al(1)-O(12)^{2}	1.876(16)

Symmetry Operators: (1) −X+1,Y,Z (2) X,−Y,−Z+1 (3) −X+1,−Y,−Z+1.

Table S2. Bond angles (°) of **TBA-1.**

**Bond Angles (°)**			
O(1)-W(1)-O(9)	91.9(6)	O(1)-W(1)-O(13)	104.8(8)
O(1)-W(1)-O(14)	98.9(8)	O(1)-W(1)-O(17)	167.2(6)
O(1)-W(1)-O(19)	102.2(6)	O(9)-W(1)-O(13)	83.3(8)
O(9)-W(1)-O(14)	87.3(7)	O(9)-W(1)-O(17)	79.0(4)
O(9)-W(1)-O(19)	165.8(5)	O(13)-W(1)-O(14)	154.7(8)
O(13)-W(1)-O(17)	83.3(6)	O(13)-W(1)-O(19)	91.5(7)
O(14)-W(1)-O(17)	71.8(6)	O(14)-W(1)-O(19)	92.0(6)
O(17)-W(1)-O(19)	87.3(5)	O(2)-W(2)-O(10)	88.2(7)
O(2)-W(2)-O(14)	100.0(8)	O(2)-W(2)-O(15)	102.3(6)
O(2)-W(2)-O(17)	166.8(6)	O(2)-W(2)-O(20)	101.4(7)
O(10)-W(2)-O(14)	85.2(6)	O(10)-W(2)-O(15)	86.4(5)
O(10)-W(2)-O(17)	80.9(5)	O(10)-W(2)-O(20)	169.6(6)
O(14)-W(2)-O(15)	155.9(7)	O(14)-W(2)-O(17)	71.9(7)
O(14)-W(2)-O(20)	89.1(6)	O(15)-W(2)-O(17)	84.5(4)
O(15)-W(2)-O(20)	95.3(5)	O(17)-W(2)-O(20)	89.1(5)
O(3)-W(3)-O(11)	99.3(6)	O(3)-W(3)-O(15)	100.8(7)
O(3)-W(3)-O(16)	98.1(7)	O(3)-W(3)-O(18)	174.9(6)
O(3)-W(3)-O(21)	97.0(6)	O(11)-W(3)-O(15)	90.6(5)

O(11)-W(3)-O(16)	90.0(6)	O(11)-W(3)-O(18)	79.6(5)
O(11)-W(3)-O(21)	163.6(5)	O(15)-W(3)-O(16)	160.8(5)
O(15)-W(3)-O(18)	84.3(4)	O(15)-W(3)-O(21)	87.5(5)
O(16)-W(3)-O(18)	76.9(5)	O(16)-W(3)-O(21)	86.5(6)
O(18)-W(3)-O(21)	84.0(5)	O(4)-W(4)-O(19)	97.7(5)
O(4)-W(4)-O(22)	100.8(8)	O(4)-W(4)-O(23)	100.6(6)
O(4)-W(4)-O(26)	174.1(5)	O(4)-W(4)-O(28)	100.4(5)
O(19)-W(4)-O(22)	84.0(7)	O(19)-W(4)-O(23)	86.3(5)
O(19)-W(4)-O(26)	80.7(4)	O(19)-W(4)-O(28)	161.6(5)
O(22)-W(4)-O(23)	157.4(7)	O(22)-W(4)-O(26)	84.6(7)
O(22)-W(4)-O(28)	89.2(7)	O(23)-W(4)-O(26)	73.7(5)
O(23)-W(4)-O(28)	93.8(6)	O(26)-W(4)-O(28)	81.7(4)
O(5)-W(5)-O(20)	97.0(7)	O(5)-W(5)-O(23)	94.2(8)
O(5)-W(5)-O(24)	103.5(8)	O(5)-W(5)-O(26)	168.8(7)
O(5)-W(5)-O(29)	98.2(8)	O(20)-W(5)-O(23)	85.8(6)
O(20)-W(5)-O(24)	87.3(5)	O(20)-W(5)-O(26)	82.3(4)
O(20)-W(5)-O(29)	164.8(6)	O(23)-W(5)-O(24)	161.7(6)
O(23)-W(5)-O(26)	74.6(5)	O(23)-W(5)-O(29)	93.2(7)
O(24)-W(5)-O(26)	87.7(5)	O(24)-W(5)-O(29)	89.0(6)
O(26)-W(5)-O(29)	82.7(5)	O(6)-W(6)-O(21)	100.3(6)
O(6)-W(6)-O(24)	98.9(7)	O(6)-W(6)-O(25)	99.0(7)
O(6)-W(6)-O(27)	171.9(6)	O(6)-W(6)-O(30)	93.8(6)
O(21)-W(6)-O(24)	88.6(6)	O(21)-W(6)-O(25)	90.5(7)
O(21)-W(6)-O(27)	84.6(6)	O(21)-W(6)-O(30)	165.0(6)
O(24)-W(6)-O(25)	162.0(6)	O(24)-W(6)-O(27)	87.7(5)
O(24)-W(6)-O(30)	84.1(6)	O(25)-W(6)-O(27)	74.3(6)
O(25)-W(6)-O(30)	92.4(6)	O(27)-W(6)-O(30)	82.0(5)
O(7)-W(7)-O(28)	101.8(7)	O(7)-W(7)-O(28)[1]	101.8(7)
O(7)-W(7)-O(31)	173.9(9)	O(7)-W(7)-O(32)	104.9(6)
O(7)-W(7)-O(32)[1]	104.9(6)	O(28)-W(7)-O(28)[1]	83.6(5)
O(28)-W(7)-O(31)	82.7(4)	O(28)-W(7)-O(32)	80.3(5)
O(28)-W(7)-O(32)[1]	151.1(4)	O(28)[1]-W(7)-O(31)	82.7(4)
O(28)[1]-W(7)-O(32)	151.1(4)	O(28)[1]-W(7)-O(32)[1]	80.3(5)
O(31)-W(7)-O(32)	71.6(4)	O(31)-W(7)-O(32)[1]	71.6(4)
O(32)-W(7)-O(32)[1]	103.1(5)	O(8)-W(8)-O(29)	103.4(8)
O(8)-W(8)-O(30)	104.1(7)	O(8)-W(8)-O(31)	168.8(7)
O(8)-W(8)-O(32)	103.3(7)	O(8)-W(8)-O(33)	109.8(8)
O(29)-W(8)-O(30)	88.9(6)	O(29)-W(8)-O(31)	83.1(6)
O(29)-W(8)-O(32)	77.2(5)	O(29)-W(8)-O(33)	146.8(6)
O(30)-W(8)-O(31)	85.0(5)	O(30)-W(8)-O(32)	151.5(5)
O(30)-W(8)-O(33)	84.4(7)	O(31)-W(8)-O(32)	68.8(4)
O(31)-W(8)-O(33)	64.0(5)	O(32)-W(8)-O(33)	93.8(5)
O(12)-P(1)-O(17)	114.6(5)	O(12)-P(1)-O(17)[1]	114.6(5)
O(12)-P(1)-O(18)	107.7(8)	O(17)-P(1)-O(17)[1]	105.8(6)
O(17)-P(1)-O(18)	106.8(5)	O(17)[1]-P(1)-O(18)	106.8(5)
O(26)-P(2)-O(26)[1]	109.6(8)	O(26)-P(2)-O(27)	111.5(5)
O(26)-P(2)-O(31)	108.9(5)	O(26)[1]-P(2)-O(27)	111.5(5)
O(26)[1]-P(2)-O(31)	108.9(5)	O(27)-P(2)-O(31)	106.3(9)
O(11)-Al(1)-O(11)[2]	89.5(5)	O(11)-Al(1)-O(11)[3]	180.0(7)
O(11)-Al(1)-O(11)[1]	90.5(5)	O(11)-Al(1)-O(12)	90.7(5)
O(11)-Al(1)-O(12)[2]	89.3(5)	O(11)[2]-Al(1)-O(11)[3]	90.5(5)
O(11)[2]-Al(1)-O(11)[1]	180.0(7)	O(11)[2]-Al(1)-O(12)	89.3(5)
O(11)[2]-Al(1)-O(12)[2]	90.7(5)	O(11)[3]-Al(1)-O(11)[1]	89.5(5)
O(11)[3]-Al(1)-O(12)	89.3(5)	O(11)[3]-Al(1)-O(12)[2]	90.7(5)
O(11)[1]-Al(1)-O(12)	90.7(5)	O(11)[1]-Al(1)-O(12)[2]	89.3(5)
O(12)-Al(1)-O(12)[2]	180.0(7)	W(3)-O(11)-Al(1)	140.1(7)
P(1)-O(12)-Al(1)	133.3(10)	W(1)-O(13)-W(1)[1]	162.2(12)
W(1)-O(14)-W(2)	125.4(10)	W(2)-O(15)-W(3)	158.0(7)
W(3)-O(16)-W(3)[1]	115.4(9)	W(1)-O(17)-W(2)	90.0(4)
W(1)-O(17)-P(1)	130.9(7)	W(2)-O(17)-P(1)	129.3(6)
W(3)-O(18)-W(3)[1]	90.0(5)	W(3)-O(18)-P(1)	125.0(4)
W(3)[1]-O(18)-P(1)	125.0(4)	W(1)-O(19)-W(4)	168.3(7)

W(2)-O(20)-W(5)	164.2(7)	W(3)-O(21)-W(6)	169.4(8)
W(4)-O(22)-W(4)^1	146.3(12)	W(4)-O(23)-W(5)	122.5(8)
W(5)-O(24)-W(6)	148.6(7)	W(6)-O(25)-W(6)^1	121.1(10)
W(4)-O(26)-W(5)	88.9(4)	W(4)-O(26)-P(2)	128.8(6)
W(5)-O(26)-P(2)	126.9(7)	W(6)-O(27)-W(6)^1	90.2(5)
W(6)-O(27)-P(2)	128.4(5)	W(6)^1-O(27)-P(2)	128.4(5)
W(4)-O(28)-W(7)	151.4(7)	W(5)-O(29)-W(8)	145.6(8)
W(6)-O(30)-W(8)	150.6(7)	W(7)-O(31)-W(8)	89.7(3)
W(7)-O(31)-W(8)^1	89.7(3)	W(7)-O(31)-P(2)	125.6(10)
W(8)-O(31)-W(8)^1	89.0(5)	W(8)-O(31)-P(2)	125.7(4)
W(8)^1-O(31)-P(2)	125.7(4)	W(7)-O(32)-W(8)	126.8(6)
W(8)-O(33)-W(8)^1	141.9(7)		

Symmetry Operators: (1) −X+1,Y,Z (2) X,−Y,-Z+1 (3) −X+1,−Y,−Z+1.

Table S3. Bond valence sums (BVSs) calculation for polyoxoanion **1.**

**BVSs**			
W(1)	6.31	O(12)	2.1
W(2)	5.89	O(13)	2.25
W(3)	5.65	O(14)	2.17
W(4)	5.72	O(15)	2.24
W(5)	6.21	O(16)	1.72
W(6)	5.95	O(17)	1.75
W(7)	6.45	O(18)	1.89
W(8)	6.33	O(19)	2.16
P(1)	5.22	O(20)	2.08
P(2)	5.49	O(21)	2.07
Al(1)	2.88	O(22)	2.07
O(1)	1.85	O(23)	1.99
O(2)	1.76	O(24)	2.06
O(3)	1.52	O(25)	1.86
O(4)	1.53	O(26)	2.04
O(5)	1.75	O(27)	1.79
O(6)	1.67	O(28)	1.98
O(7)	1.9	O(29)	1.95
O(8)	1.7	O(30)	2.05
O(9)	0.54	O(31)	2.04
O(10)	0.63	O(32)	2.16
O(11)	1.53	O(33)	2.80

© 2019 by the authors. Submitted for possible open access publication under the terms and conditions of the Creative Commons Attribution (CC BY) license (http://creativecommons.org/licenses/by/4.0/).
